# Varicocele Time-dependently Affects DNA Integrity of Sperm Cells: Evidence for Lower *In vitro* Fertilization Rate in Varicocele-positive Rats

**Published:** 2011-12-22

**Authors:** Mazdak Razi, Rajab-Ali Sadrkhanloo, Hassan Malekinejad, Farshid Sarafzadeh-Rezaei

**Affiliations:** 1 Department of Comparative Histology and Embryology, University of Urmia, Urmia, Iran; 2 Department of Pharmacology and Toxicology, Faculty of Veterinary Medicine, University of Urmia, Urmia, Iran; 3 Department of Clinical Science, Division of Surgery, University of Urmia, Urmia, Iran

**Keywords:** DNA Damage, *In vitro* Fertilization, Oxidative Stress, Varicocele

## Abstract

**Background:**

We designed this study to clarify how varicocele can time-dependently affect sperm morphological parameters and DNA integrity. In this study, we intend to estimate the effect of various periods of varicocele on the *in vitro* fertilization (IVF) rate in rats.

**Materials and Methods:**

In this experimental study, left varicocele were induced as the test group (n=18) which was further sub-divided into three groups based on the study termination time (4, 6 and 8 months after varicocele induction). The control-sham group (n=6) consisted of rats who received no treatment. Repopulation index (RI), tubular differentiation index (TDI), sperm viability and motility, morphological maturity, chromatin integrity and ability to undergo IVF were assessed. In addition, the potential impact of varicocele on serum total antioxidant capacity (TAOC) and total thiol molecules (TTM) were examined.

**Results:**

Histological results showed that varicocele negatively influenced TDI and RI. All sperm morphological parameters were lower than those in the control-sham group. DNA damage was severely and time-dependently substantiated in all test groups. Varicocele significantly reduced the ability of sperm derived from varicocele rats to undergo IVF. Serum TAOC and TTM levels reduced in a time-dependent manner. Right testes varicocele-induced rats showed remarkably less damaged profile for all investigated parameters compared to the left testes varicocele.

**Conclusion:**

Our data suggested that experimentally induced varicocele negatively impacted sperm maturation and chromatin integrity in a time-dependent manner. This consequently caused a remarkable reduction in IVF ability. The detrimental effect of varicocele may be attributed to the significant reduction of antioxidant capacity of the serum.

## Introduction

Varicocele is defined by abnormal tortuosity and pampiniform plexus venous dilation within the spermatic cord and is the most common surgically correctible cause of male infertility (1, 2). According to several clinical reports, varicocele is observed in 10-20 % of the general male population, 35-40% of men with primary infertility and up to 80% of men with secondary infertility (3, 4). Varicoceles are progressive, often appearing at puberty and more commonly (90%) located on the left side (5). Despite numerous studies that have focused on varicocele, the exact mechanism(s) by which varicocele induces testicular degeneration, dysfunction and finally infertility have not completely understood. The suggested mechanisms include reflux of toxic metabolites from the adrenal and/ or renal origin, impairment of the hypothalamicgonadal axis, venous stasis leading to testicular hypoxia and temperature elevation in the testicles (6, 7). However, it has long been recognized that left-sided varicoceles can have bilateral effects (2, 8). The pathophysiologic influence of varicocele differs depending on the time and exact mechanism by which this deficiency affects semen parameters. Often, varicocele results in a generalized degeneration of sperm production (9, 10).

A relationship between infertility and the generation of reactive oxygen species (ROS) has recently been established and widely studied (11-13). According to previous reports following different toxicological, iatrogenic and genetically-induced degenerations, the mitochondria and plasma membrane of morphologically abnormal spermatozoa produce ROS through the nicotinamide adenine dinucleotide phosphate-dependent and nicotinamide adenine dinucleotide-dependent oxidoreductase systems (6, 14).

In addition, oxidative stress has been shown to affect the integrity of the sperm’s genome by causing high frequencies of single- and double-strand DNA breaks, often detected in the ejaculates of infertile men (15-17). Thus, the primary aim of the present study was to evaluate the concomitant effect of both left and right testicular varicocele on spermatogenesis. Secondly, we aimed to estimate semen quality, sperm DNA fragmentation and the *in vitro* fertilizing ability of sperm at various times following varicocele induction. We also sought to analyze serum total antioxidant capacity (TAOC) and total thiol molecules (TTM) in order to clarify any pathological alterations in serum antioxidant capacity as well as to illustrate the relationships of these factors with testicular degeneration and semen quality during different intervals post-varicocele induction. In the present study, rats were selected as a laboratory model for humans.

## Materials and Methods

### Animals

In this experimental study,twenty-four mature male Wistar rats, 10 weeks old that weighed 200 ± 14 g were used. The rats were purchased from the Animal Resources Center, Faculty of Veterinary Medicine, Urmia University, Urmia, Iran and were acclimatized in an environmentally controlled room (temperature: 20-23°C and a 12 hours light/12hours dark schedule). Food and water were available ad libitum. In this study, all experiments conducted on the animals were in accordance with Urmia University’s guidance from the Ethical Committee for Research on Laboratory Animals. Following one week acclimation, the animals were assigned to four groups (n=6) of control-sham and test groups. The test group was further divided into 3 subgroups according to the study termination (4, 6 and 8 months) after varicocele induction.

### Varicocele induction

In the test groups, the left varicocele was induced as previously reported (18). Briefly, following anesthesia with 5% ketamine (40 mg/kg, i.p.) and 2% xylazine (5 mg/kg, i.p.), the diameter of renal vein was reduced to 1 mm and a left renal vein ligation was performed medial to the junction of the adrenal and spermatic veins. Then, the anastomotic branches between the left testicular and left common iliac vein were ligated. The control-sham group, while under anesthesia, only underwent a simple laparatomy with no ligation.

### Histological analyses

Animals were euthanized on 4, 6 or 8 months following varicocele induction, by a special CO2 device. The testes were dissected out, fixed in 10% formalin fixative for histological investigations and subsequently embedded in paraffin. Sections (5-6 μm) were stained with Iron-Weigert (Pajohesh Asia, Iran) for detection of germinal cell nuclei in the testis. The sections were analyzed under light microscope at two magnifications (×400 and ×1000).

### Tubular differentiation index

To estimate tubular differentiation index (TDI), the percentage of seminiferous tubules (STs) that contained more than three layers of differentiated germinal cells from spermatigonia type A were considered negative. Those which showed more than three and/or four layers were considered TDI positive. From each sample, 20 sections (6 μm) were prepared and analyzed.

### Repopulation index

We used the repopulation index (RI) to calculate the ratio of active spermatogonia (spermatogonia type B) with light nuclei to inactive spermatogonia (spermatogonia type A) with dark nuclei as seen by Iron-Weigert staining in STs from 20 prepared sections per sample, as described earlier (19, 20).

### Epididymal sperm count, viability, nucleus maturity and quantitative sperm motility

Epididymides were carefully separated from the testicles under x20 magnification with a stereo zoom microscope (Model TL2, Olympus Co., Tokyo, Japan). The epididymis was divided into 3 segments: head, body and tail. The epididymal tail was trimmed and minced in 5 mL Hams F10 medium. After 20 minutes, the ground epididymal tissue was separated from the released spermatozoa and counted. Smears were prepared by eosinnegrosin to evaluate dead, abnormal and morphologically immature sperm (MIS). Sperm which stained red were considered non-viable; those with cytoplasmic residuals were considered morphologically immature.

Aniline blue staining was performed in order to evaluate sperm nuclear maturity (or protamine deficiency). The proportion of motile spermatozoa was determined by counting 100 cells in randomly selected fields for each sample at 37°C (21).

### Epididymal sperm DNA fragmentation and double- strand DNA breaks

The comet assay (Cat No. 1435723/34, Sigma Co., St. Louis, MO, USA) was used to investigate DNA fragmentation A ×40 objective device (Olympus, Germany) was linked to an epi-fluorescent microscope (Model GS7, Nikon Co., Japan) and used for visualization of DNA damage. Light green spots with typical tails were considered to be damaged DNA and spots with no tails were marked as normal DNA.

To evaluate DNA double strand breaks, an acridine orange staining kit (Sigma Co., St. Louis, MO, USA) was used. Samples were analyzed by epifluorescent microscope (Model GS7, Nikon Co., Japan). Sperm samples for acridine orange staining were provided as previously described (22).

### Sperm processing for in vitro fertilization

Samples that contained spermatozoa were prepared from the sperm suspensions as mentioned earlier. The samples were incubated at 37°C under 5% CO2 (CO2 Incubator, LEEC, England) for 3 hours. Then, as previously described, 0.1 ml from superficial sperm and/or 0.1 ml from sediment sperm of suspensions in one tube were added to 150 μl of tissue culture medium (TCM) that contained the oocytes (23).

### Collection of oocytes and insemination

Mature female rats were injected subcutaneously with 7.5 IU pregnant mare’s serum hMG (hMG, Netherlands) 48 hours prior to an i.p. injection of 100 IU human chorionic gonadotropin hCG (Teikoku Zohki Co., korea). Rats were euthanized with a special CO2 device 24 hours after hCG injection. The oviducts were removed and the ampullar portion was placed into a plastic dish that contained PBS (pH=7.2). The oocytes that were in cumulus masses were dissected out of the oviducts and introduced into tissue culture medium 199 (TCM 199, Sigma Co., USA). A drop of medium with 2 oocytes was allocated with a 10 μl sperm suspension (total: 80,000 sperm). For each animal, a total of 20 oocytes were divided into 10 drops.

### Assessment of serum total antioxidant capacity

To determine the effect of varicocele on oxidative stress, total antioxidant capacity (TAOC) of the control-sham and test groups were measured. The assessment was performed based on the ferric reduction antioxidant power (FRAP) assay (19). Briefly, at low pH (acetate buffer, 300 mM, pH 3.6), reduction of the FeIII-TPTZ complex to the ferrous form produces an intensive blue color measurable at 593 nm. The intensity of the complex following addition of the appropriate volume of serum to the reducible solution of FeIII-TPTZ is directly related to the total reducing power of the electron donating antioxidant. An aqueous solution of FeII (FeSO4.7H2O) and appropriate concentration of freshly prepared ascorbic acid are used as blank and standard solutions, respectively.

### Measurement of serum total thiol molecules

The total sulfhydryl level in serum was measured according to Hu and Dillared method (19). In short, 0.2 ml serum was added to 0.6 ml Tris-EDTA buffer (Tris base 0.25 M, EDTA 20 mM, pH= 8.2) followed by the addition of 40 μl DTNB (10 mM in pure methanol) in a 10 ml glass test tube. The final volume of the mentioned mixture was made up to 4.0 ml by extra addition of methanol. After incubation for 15 minutes at room temperature, the samples were centrifuged at 3000 x g for 10 minutes and ultimately the absorbance of the supernatant was assessed at 412 nm.

### Statistical analyses

Statistical analyses were performed on all numerical data by two-way ANOVA and SPSS software version 13.00. All values were expressed as mean ± SD. P<0.05 was considered statistically significant.

## Results

### Histological analyses

**Fig 1:**
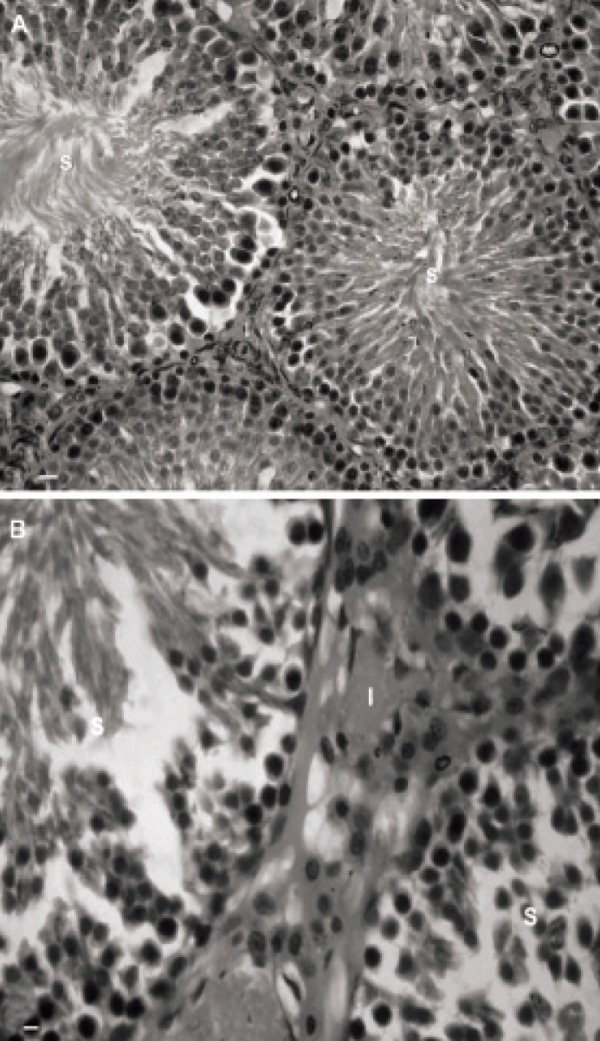
Photomicrograph of rat testis. A. Control-sham group. The seminiferous tubules with normal cellular junction (S) and interstitial connective tissue without any edema are seen (I). B. Left varicocelized testis. The dissociated germinal epithelium in seminiferous tubules (S) with edema in the interstitial connective tissue is observed (I). Iron-Weigert staining, (A × 100 and B × 400).

Light microscopic analyses of the seminiferous tubules (STs showed most tubules with germinal epithelium dissociation and disruption of the cellular junction between sertoli and spermatogenesis cells. More than 33.50 ± 5.80% of STs in the left testes and 21.25 ± 2.21% in the right testes .showed dissociated epithelium after 4 months of varicocele induction. This phenomenon increased over time; after 6 months, 41.50 ± 3.10% of STs, and at 8 months, 52.25 ± 1.70%, of STs demonstrated cellular dissociation in the left testes. The right testes of the test groups showed 34.25 ± 3.09% and 38.12 ± 1.93% dissociation in the germinal epithelium of tubules at 6 and 8 months after varicocele initiation, respectively. No histological alterations were noted in the control-sham group and the STs manifested with normal cellular junction (Fig 1).

**Fig 2:**
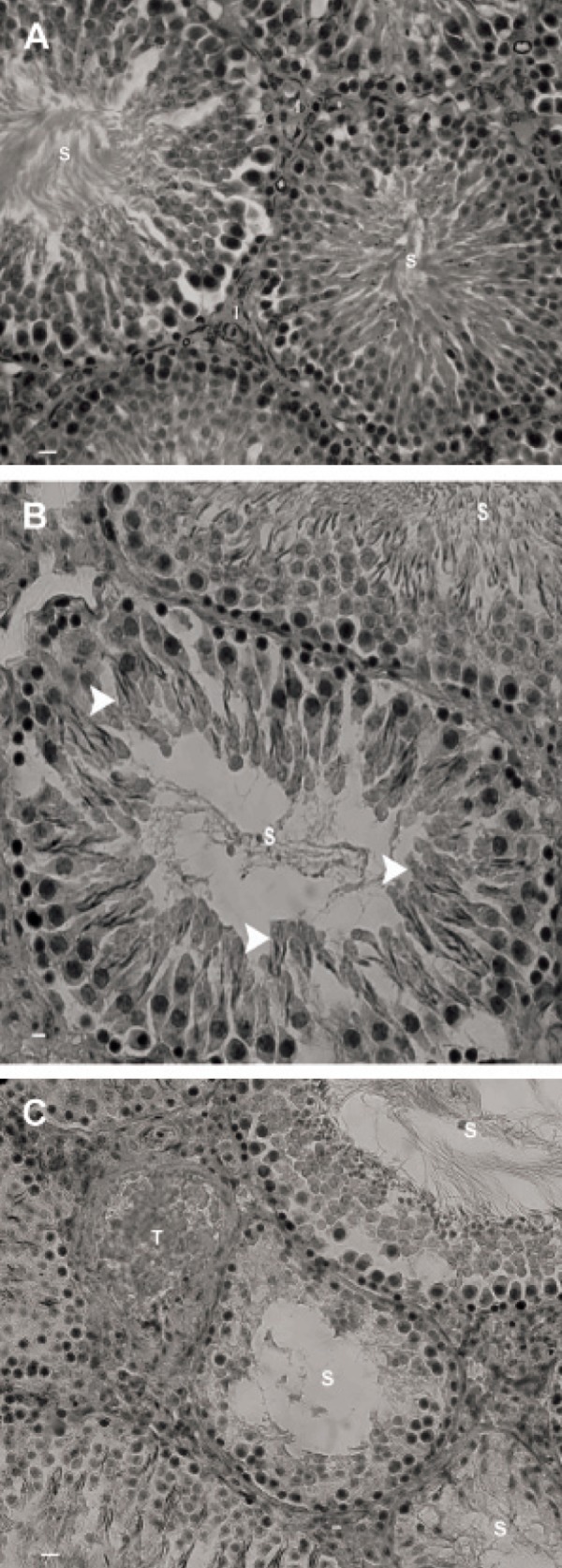
Photomicrograph of rat testis. A. Control-sham group. Note the seminiferous tubules with normal cellular junction (S) and the interstitial connective tissue with no edema (I). B. Right testes of varicocelized rats. Note the seminiferous tubules with negative tubular differentiation index (S). The spermatogenesis regions (arrowheads) are indicative of early maturation from the previous cycle. C. Left testis of varicocelized group. The seminiferous tubules (S) are completely depleted with no detectable spermatic maturation. Vascular thrombosis (T) is also observed in interstitial connective tissue. Iron-Weigert staining, (A × 100; B × 400 and C × 400).

Histological observations demonstrated that most STs had negative TDI, which further deteriorated with time in the test groups. The majority of STs showed depletion and reduction of germinal cell layers after 8 months. Right testes of the test animals showed relatively moderate destruction in STs when compared to the left testes. No histological abnormality was observed in the control-sham group (Fig 2).

**Fig 3:**
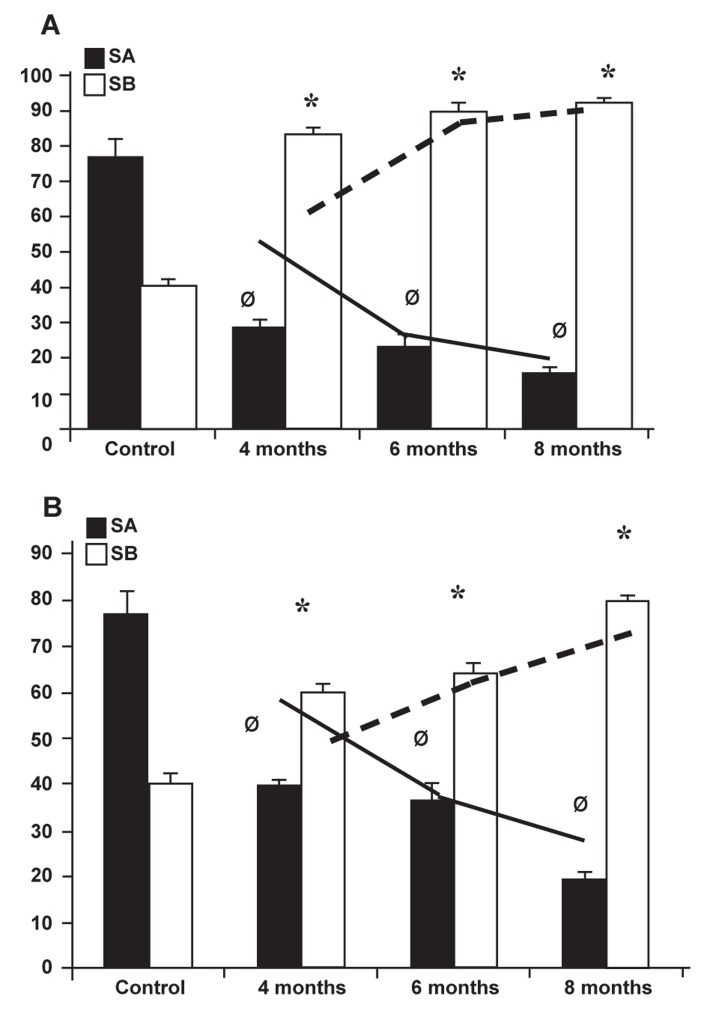
Effect of varicocele on repopulation index in: (A) Left testis and (B) right testis. Repopulation index percentage of spermatogonia type A reduced over time in all varicoceleinduced rats and the spermatogonia type B increased by the time. Ø and stars indicate significant differences (p ≤ 0.05) between data for spermatogonia types A and B, respectively. All data are presented as mean ± SD.

Special nucleus staining (Iron-Weigert) was performed in order to differentiate inactive cells (spermatogonia type A) from active cells (spermatogonia type B). The results showed that after varicocele induction the RI was negative in most test group STs and the number of STs with negative RI increased over time in both the right and left testes. Although pathologic RI was present in both testes, the right testes of the test animals showed lower negative RI in comparison to the left (Fig 3-A and 3-B). Further analyses showed some STs with maturation arrest (MA) in the test groups that were substantiated by time. The quantitative data for TDI and MA are shown in table 1.

**Table 1:** Effect of varicocele on mean average of tubular differentiation index and maturation arrest percentage in the right and left testes of test and control-sham groups


Tubular differentiation index (TDI, %)		
Groups	Left testicle	Right testicle

**Control-sham**	2.25 ± 1.00	2.75 ± 0.95
**4 months**	23.25 ± 3.59^*b'^	14.5 ± 3.31^*a'^
**6 months **	33.5 ± 3.41^*b^	23.25 ± 3.59^*b'^
**8 months**	37.87 ± 2.65^*c^	32.50 ± 3.10^*c'^
**Maturation arrest (MA%)**		
**Control-sham**	0.7±0.44	0.75 ± 0.28
**4 months**	33.6±3.57^*a^	25.50 ± 1.91^*a'^
**6 months**	39.2±1.92^*^	38.5 ± 1.29^*^
**8 months**	44.6±3.20^*b^	40.25 ± 1.70^*b'^


Stars indicate significant differences (p≤0.05) between the control-sham and test groups in the same column. Letters in the same rows represent significant differences (p≤0.05) between the left and right testes in same months. All data are presented as mean ± SD.

### Sperm count and morphology

**Fig 4:**
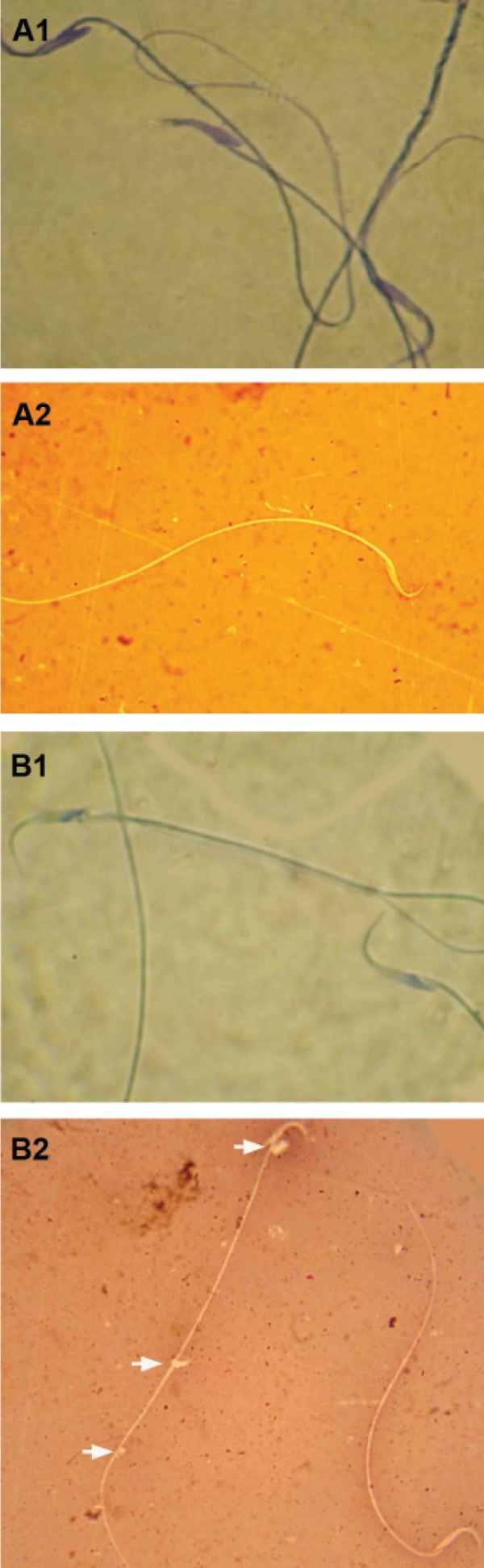
Light microscopic architecture from sperm; A1; Abnormal sperm with dense blue stained mature nucleus, A2; Normal sperm with unstained cytoplasm in head section, B1; Normal sperm with light stained immature nucleus, and B2; Abnormal sperm; note the sperm in the left side of the figure with cytoplasmic droplet (arrows) and the dead sperm with eosin-stained cytoplasm (below, right hand side). Aniline-blue (A-1, B-1) and eosin-negrosin (A-2, B-2) stainings, (× 400).

Hemocytometric evaluations for sperm count showed that after varicocele induction the sperm number reduced significantly (p<0.05); this reduction increased with time. Eight month varicoceleinduced cases manifested severely decreased sperm count; one of the six rats in this group was azoospermic. The right testes of varicocele-induced animals showed relatively higher sperm counts in all test groups when compared to the left testes. Aniline blue staining for sperm nucleus maturity revealed that the ratio of immature sperm with light stained nuclei increased remarkably in varicocele-induced animals (Fig 4A). The rats that suffered from varicocele for 8 months manifested higher numbers or percentages of immature sperm when compared with the other test groups. Furthermore, the sperm with cytoplasmic droplets were considered MIS. Observations demonstrated that the number of sperm with cytoplasmic droplets increased significantly (p<0.05) in varicocele-induced groups. Accordingly, 8 month varicocele-induced rats had the highest MIS value. In comparison to the left testes, the right testes showed a lower rate of immature sperm. A comparison of the results between sperm collected from the right testes of the varicocele-induced group with the control-sham group revealed that MIS was significantly (p<0.05) higher in right testes samples (Fig 4B).

Light microscopic analyses for sperm motility showed that the percentage motility decreased over time in varicocele-induced rats. Sperm delivered from the right testes of varicocelized animals exhibited relatively higher sperm motility in comparison to the left testes.

According to the results from eosin-negrosin staining, there was lower sperm viability in varicocele- induced rats, with the highest sperm mortality seen in the 8 month varicocelized animals. The percentage of viable sperm was also higher in the right varicocele group when compared with the left varicocele group. Data for sperm count and morphology are presented in table 2.

**Table 2:** Effect of varicocele on: mean average for sperm count (S/C), sperm normality (S/M), sperm viability (S/V), morphological immature sperm (MIS) and nuclear immature sperm (NIS) in the different test groups and control-sham group


Left testes				
Parameters	Control-sham	4 months	6 months	8 months

**S/C (×10^6^)**	72.50 ± 3.78	42.50 ± 2.08^*a^	38.00 ± 2.16^*b^	31.75 ± 2.36^*c^
**S/M-%**	92.25 ± 1.70	91.00 ± 1.41^*a^	39.75 ± 1.70^*b^	33.75 ± 2.62^*c^
**S/V- %**	92.33 ± 2.08	57.34 ± 5.03^*a^	44.66 ± 4.16^*b^	32.34 ± 2.51^*c^
**MIS-%**	16.20 ± 4.43	50.80 ± 1.64^*a^	59.20 ± 1.66^*b^	70.20 ± 1.78^*c^
**NIS-%**	9.25 ± 0.95	48.75 ± 2.21^*a^	57.00 ± 1.82^*b^	62.25 ± 1.70^*c^
**Right testes**				
**S/C(×10^6^)**	73.75 ± 3.30	54.50 ± 3.41^*a'^	43.75 ± 2.06^*b'^	37.25 ± 1.70^*c'^
**S/M-%**	91.25 ± 1.50	56.75 ± 1.70^*a'^	44.00 ± 3.65^*b'^	36.00 ± 4.08^*c'^
**S/V-%**	92.60 ± 3.05	70.66 ± 3.05^*a'^	61.34 ± 3.05^*b'^	44.67 ± 4.16^*c'^
**MIS-%**	15.80 ± 5.63	48.60 ± 1.67^*a'^	55.60 ± 2.60^*b'^	62.80 ± 2.77^*c'^
**NIS-%**	9.26 ± 1.70	42.50 ± 2.51^*a'^	46.25 ± 2.36^*b'^	53.72 ± 3.30^*c'^


Stars indicate significant differences (p<0.05) between the control-sham and test groups in the same row. Different letters in the same columns are significant differences (p<0. 05) between the right and left testes in the same month. All data are presented as mean ± SD.

**Table 3:** Effect of varicocele on mean average for DNA fragmentation and DNA double-strand breaks in the test groups and control-sham group


DNA fragmentation (comet assay,%)		
Groups	Left testes	Right testes

**Control-sham**	4.20 ±0.83	4.40±1.14
**4 months**	35.0±2.30^*a^	30.60±1.14^*b^
**6 months**	41.20±1.30^*a^	37.00±0.70^*b^
**8 months**	53.40±3.28^*a^	48.80±1.30^*b^
**Maturation arrest (MA%)**		
**Control-sham**	3.00±0.70	3.20±0.70
**4 months**	34.60±3.43^*a^	28.20±1.78^*b^
**6 months**	39.80±1.48^*a^	34.00±3.39^*b^
**8 months**	45.80±1.92^*a^	41.20±1.78^*b^


Stars indicate significant differences (p < 0.05) between the control-sham and test groups in the same column. Letters in the same rows indicate significant differences (p ≤ 0.05) between the left and right testes in the same months. All data are presented as mean ± SD.

### Sperm DNA fragmentation and double-strand breaks

**Fig 5:**
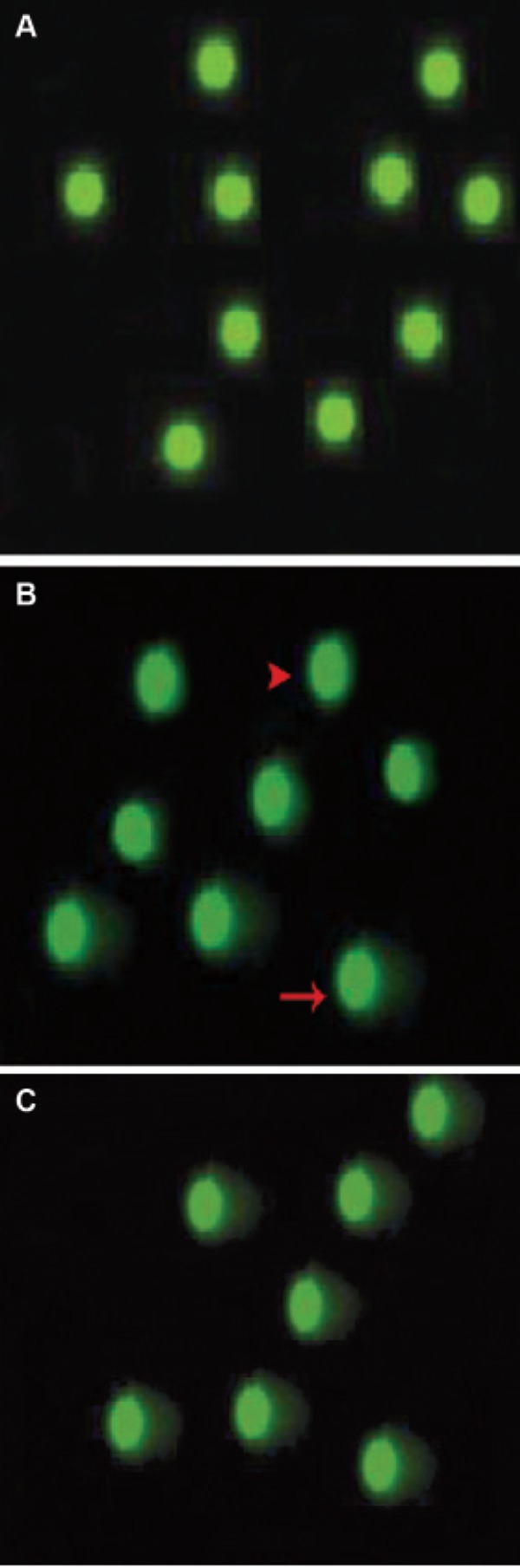
Epi-fluorescent architecture of rat sperms by Comet assay. A. Sperms from control group; the green spots without any tails are normal sperm. B. Sperms from right testes; The green spots without tails (arrowhead) and spots with tails (arrow) indicate the DNA fragmentation. In panel B both normal and fragmented DNA are seen in sperms collected from the right testes of varicocelized rats, C. Sperms collected from the left testes of varicocelized rats with intensive DNA fragmentation; (Comet assay technique, × 100).

Results from the comet assay showed an elevated ratio of sperm DNA fragmentation in the varicoceleinduced animals that increased over time. Sperm from the left varicocelized testes had higher DNA fragmentation when compared with the right testes in the same animals and the control-sham group (Fig 5).

**Fig 6:**
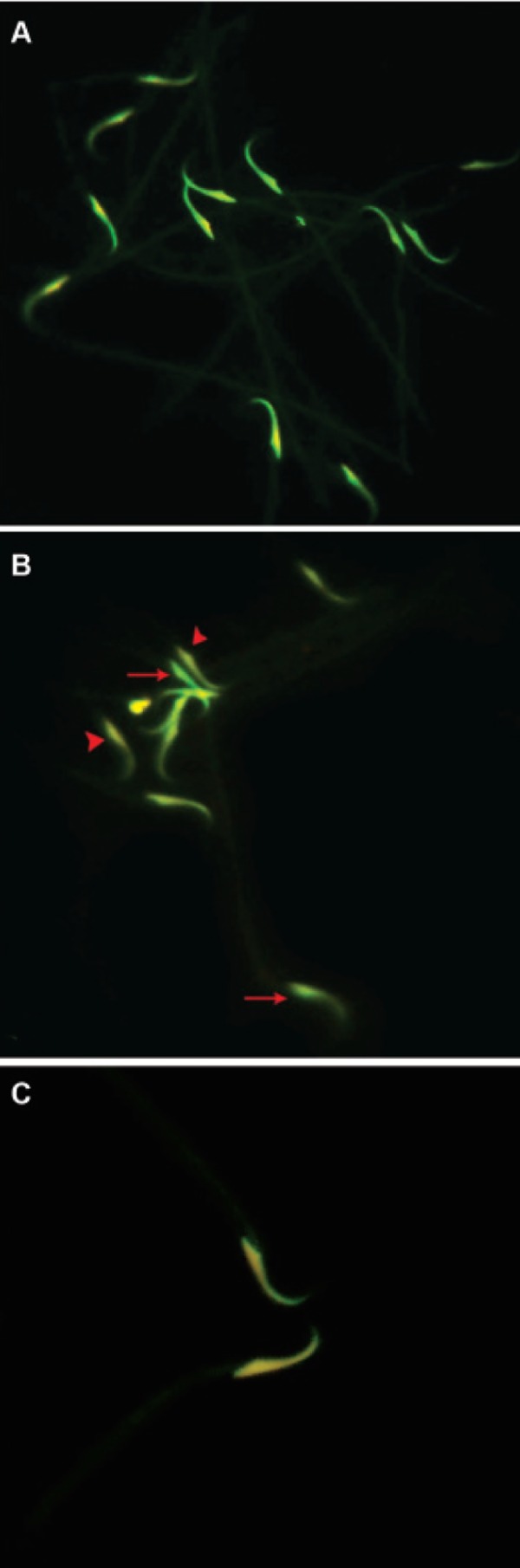
Effect of varicocele on double strand DNA in rat sperm. A. Epi-fluorescent architecture of the control-sham group. The light green stained nuclei are indicative of the normal double-strand DNA in this group. B. Light microscopic architecture of sperm from right testes of varicoceleinduced rats. The normal sperm with light green stained nucleolus (arrows) and sperm with damaged DNA with light yellow stained nucleolus (arrowheads) are presented. C. Light microscopic architecture of sperm from left testis of varicocele-induced rat, the sperm with light yellow stained nucleolus represent remarkable DNA damage (Acridineorange staining, × 400).

Acridine-orange staining showed that the number of sperm with double-strand DNA breaks was significantly (p<0.05) higher in the varicocelized groups with the highest rate of DNA breaks seen in rats with 8 months varicocelized testes. The percentage of DNA breaks was significantly (p<0.05) lower in sperm from the right testes of varicocelized animals when compared to samples from the left testes (Fig 6). The numerical data for sperm DNA fragmentation and breaks are presented in table 3.

**Table 4:** Effect of varicocele on means average of 4- and 8-cell embryos, moru- la and blastocysts after in vitro insemination of oocytes with superficial and sediment sperm contents of the right and left testes from the test and control- sham groups


Groups	4-cell embryo	Left testes8-cell embryo	Morula	Blastocyst

**Control-sham**	27.80 ± 2.94	24.20 ± 0.83	22.06 ± 1.67	20.50 ± 1.04
**4 months**	13.66 ± 2.25^*a^	12.20 ± 1.64^*b^	11.40 ± 0.84^*c^	11.20 ± 0.83^*d^
**6 months**	5.50 ± 1.16^*^	5.80 ± 1.30^*^	5.60 ± 1.07^*c^	5.40 ± 2.50^*d^
**8 months**	4.83 ± 0.75^*a^	3.40 ± 1.34^*b^	3.00 ± 0.70^*c^	2.80 ± 0.83^*d^
**Sediment sperm content from left testes (%)**				
**Control-sham**	27.16 ± 1.47	24.40 ± 1.14	22.60 ± 1.14	19.83 ± 0.75
**4 months**	11.80 ± 1.48^*a'^	10.60 ± 1.14^*b'^	9.20 ± 0.83^*c'^	9.00 ± 1.41^*d'^
**6 months**	5.00 ± 1.41*	4.20 ± 1.09^*^	4.00 ± 0.70^*c'^	3.80 ± 0.44^*d'^
**8 months**	1.40 ± 0.89^*a'^	1.30 ± 0.83^*b'^	1.20 ± 1.09^*c'^	0.60 ± 0.54^*d'^
**Superficial sperm content from right testes (%)**				
**Groups**	4-cell embryo	8-cell embryo	Morula	Blastocyst
**Control-sham**	27.60 ± 1.81	24.80 ± 1.48	20.80 ± 0.83	19.80 ± 1.14
**4 months**	14.00 ± 1.00^#e^	12.37 ± 1.52^#f^	12.00 ± 1.00^#g^	11.57 ± 0.95^#h^
**6 months**	8.33 ± 1.15^#e^	8.00 ± 1.00^#f^	7.66 ± 0.57^#g^	7.00 ± 0.81^#h^
**8 months**	6.34 ± 0.57^#e^	5.25 ± 0.50^#f^	4.60 ± 0.57^#g^	4.25 ± 0.95^#h^
**Sediment sperm content from right testes (%)**				
**Control-sham**	27.33±1.03	24.83 ± 1.47	21.50 ± 1.04	19.84 ± 1.47
**4 months**	13.25±1.25^#e'^	12.10 ± 0.84^#f'^	10.62 ± 0.47^#g'^	9.98± 0.14^#h'^
**6 months**	7.50±1.29^#e'^	6.75 ± 0.95^#f'^	5.70 ± 0.94^#g'^	5.00 ± 0.08^#h'^
**8 months**	4.50±1.29^#e'^	9.25 ± 0.95^#f'^	2.20 ± 0.54^#g'^	2.25 ± 0.51^#h'^


Stars and # indicate significant differences (p < 0.05) between data in the same column with control-sham rats; different letters indicate significant differences (p < 0.05) between data of superficial sperm and sediment sperm contents. All data are presented as mean ± SD. A total of 20 oocytes were used for each sperm sample and the percentages in the table were calculated from 20 oocytes.

### Varicocele influences the rate of in vitro fertilization

**Fig 7:**
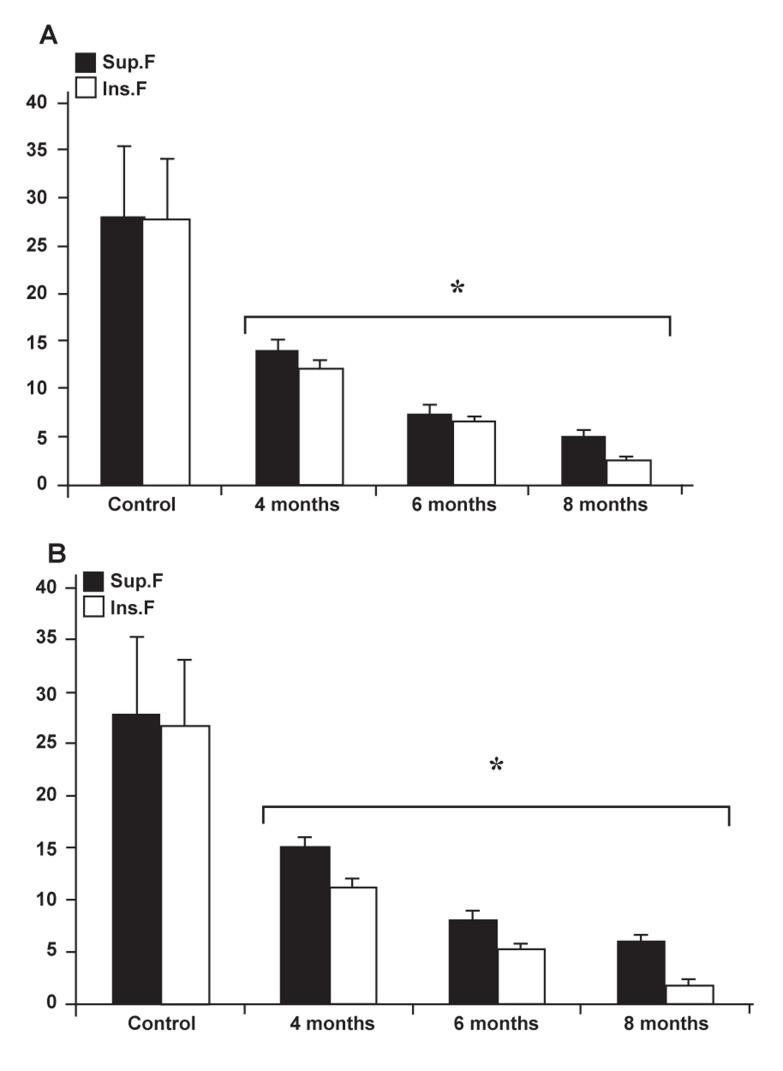
Effect of varicocele on IVF and 2-cell embryo after invitro fertilization in the control and varicocele-induced rats: A. Left testicle data, B. right testicle data. Stars indicate significant differences (p ≤ 0.05) between all data for different months following varicocele induction with each test group and the control group. All data are presented as mean ± SD.

The results for *in vitro* fertilization(IVF) of oocytes by sperm collected from the right and left testes of varicocelized animals were remarkably lower than the control-sham group (Fig 7A, B). The considerable point was that most of the 2-cell embryos arrested at the first cleavage stage and did not continue further in the varicocelized group. This phenomenon was at its highest rate in samples from the left testes of the varicocelized animals for 8 months. The results for sperm collected from the upper side of the tube (superficial sperm) in terms of progress in cell division were significantly (p<0.05) higher than those collected from the lower side of the tube (sediment sperm). A comparison of the values for fertilization rates between all test groups indicated that the highest value belonged to oocytes exposed to sperm from the right testes of varicocelized animals for 4 months. Therefore, in varicocelized animals the rate of the morula and blastocysts phases compared to the control-sham group declined significantly (p<0.05). The data for IVF rates are presented in table 4.

### Alterations in TAOC and TTM

**Fig 8:**
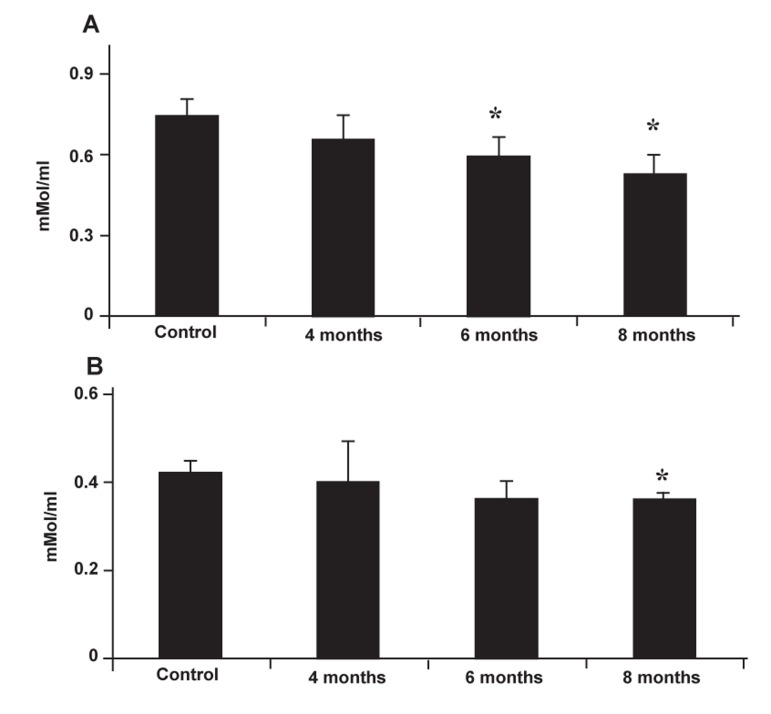
Effect of varicocele on the serum level of TAOC (A) and TTM (B); Stars indicate significant differences (p ≤ 0.05) between control and test groups. All data are presented as mean ± SD.

Results indicated that both TAOC and TTM significantly (p<0.05) decreased after varicocele induction for 4, 6 and 8 months compared to the control-sham group. Accordingly, animals in the 8 month varicocele-induced group showed remarkable diminished antioxidant capacity of serum (Fig 8 A, B).

## Discussion

In the present study we showed that in varicoceleinduced rats there were early MI, negative TDI and RI due to disrupted spermatogenesis in both the left and right testes. The negative impact of varicocele increased over time. Our further analyses illustrated that following varicocele-induction, in addition to a reduction in sperm motility, sperm protamine-histone transition, DNA and plasma membrane integrity were impaired with a subsequent loss of motility that increased over time. Ultimately, our results showed that the quality of sperm content reduced with time, which in turn reduced the IVF rate.

Varicocele is a disorder with an incidence of 10% to 20% in the general population (24) that is commonly diagnosed among men with infertility (25). Various factors associated with varicocele may lead to DNA damage, including: heat stress (26, 27), androgen deprivation (6, 28), exposure to toxic agents (29, 30), testicular hypoxia (31, 32) and increased oxidative stress (7, 33). In the present study, we have used specific methods to clarify the effect of the duration of varicocele and consequently the probable effect of varicocele-induced oxidative stress on sperm morphology, maturation and DNA integrity. We also sought to identify the IVF ability of these sperm during different time periods.

According to our findings, varicocele does not exert its pathologic effect immediately, but rather it takes time to see the various pathological features associated with varicocele. In many pathological conditions, apoptosis of germinal cells in STs is the main cause of germ cell loss and this impairment can also influence sertoli cells (34-37). According to several independent reports, high scrotal temperatures and/or elevated oxidative stress in spermatogenesis results in apoptosis, thus leading to remarkable cellular depletion in STs (38-40). Our findings are in concordance with these reports. We have illustrated that after varicocele initiation, the RI ratio in STs is negative, which indicates severe degeneration in spermatogonia cells. Furthermore, our light microscopic analyses have shown an intensive cellular disruption and substantial STs depletion in the majority of STs in varicocelized animals as well as severe damage in sertoli cells. Interestingly, due to hypospermtogenesis and high MI occurrence, the total sperm content and percentage of sperm maturity significantly reduced in varicocele cases over time.

In damaged spermatogenesis, the cytoplasmic extrusion mechanisms do not function normally (19). In this condition the released spermatozoa from the germinal epithelium carry surplus residual cytoplasm and can be counted as immature and functionally defective spermatozoa (19, 20, 41, 42). It is well documented that the level of ROS production correlates negatively with sperm quality in the original semen (43, 44).

In the current study, samples obtained from 4 month varicocelized rats contained MIS with cytoplasmic droplets (residual cytoplasm). The rate of immature sperm occurrence was found to correlate with imbalance in oxidative defense of the 8 month varicocelized rats. Therefore, the rate of immature sperm was higher in the samples with lowest TTM and TAOP. Evidence has shown that any disruption in the physiological function of the cytosolic enzyme glucose-6-phosphate-dehydrogenase, the enzyme that mediates some physiological bioactivities involved in sperm maturity, can influence sperm maturity processes in varicocele patients (42). Interestingly in this study, after 4 and 6 months serum TAOC and thiol molecules decreased in comparison to control cases, but this reduction was not statistically significant (p>0.05). The intensive and remarkable reduction in TTM and TAOP levels as biomarkers of oxidative stress occurred 8 months following varicocele induction. Sperm immaturity and abnormality may have significantly increased independently to serum antioxidant capacity from 4 months following varicocele induction, which progressed over time. Thus, these findings may suggest that the pathological effect observed in early varicocele is independent of reduced sperm antioxidant capacity while the later effects of varicocele may be increased due to reduced serum antioxidant capacity. Thus our results confirm the fact that in varicocelized animals the extent of damage caused by ROS on sperm parameters depends not only on the nature and amount of ROS involved but also on duration of ROS exposure. According to earlier studies, increased ROS formation has been correlated with a reduction of sperm motility (45-47). The link between ROS and reduced motility can be explained by two hypotheses. First, a wave of events that result in an intensive decrease in axonemal protein phosphorylation and sperm immobilization, and secondly, free radicals such as H2O2 which can diffuse across the membranes into cells and inhibit the activities of enzymes such as glucose 6-phosphate dehydrogenase (48, 49). Our observations have demonstrated that after 8 months the varicocele-induced rats manifested a high percentage of immotile sperm. This may have occurred because spermatozoa are particularly susceptible to ROS stress since their plasma membranes are enriched with unsaturated fatty acids, particularly decosohexaenoic acid with six double bonds per molecule. In order to exactly identify the rate of sperm plasma membrane damage, eosin-negrosin staining was performed which confirmed this hypothesis showing an increased number of sperm with defective plasma membranes.

A number of studies have suggested that the presence of spermatozoa with damaged DNA may be the result of impaired chromatin packing or indicative of apoptosis (50, 51). There are external factors such as transitional changes in the concentration of trace elements that promote oxidative stress impact and DNA damage. The protamination status of chromatin can remarkably protect sperm from these damages (52, 53). Protamines may act as protective elements by sequestering trace elements capable of promoting the fragmentation of sperm DNA (54). Our special staining for protamine showed that after varicocele induction, the protamine-histone transition was impaired which may have partially accounted for the extensive DNA damage observed in poorly packaged spermatozoa of the varicocelized rats. On the other hand, results from the comet assay and acridine orange stainings were in accordance with these findings and showed an elevated sperm DNA fragmentation and damage in varicocele-induced rats.

Reports have indicated that disorders which have resulted in failure of epididymal sperm maturation also impair sperm fertilizing ability (40, 48, 50, 55). It is well known that during the epididymal transition sperm acquires it ability to present forward motility, undergo capacitation and finally penetrate the zona pellucida (21). Our observations have revealed that during long time varicocele, MIS increased remarkably.

According to our results the unsaturated fatty acids in plasma membrane of sperms were severely damaged after varicocele induction. These unsaturated fatty acids are essential for fluidity for the plasma membrane in order to participate in membrane fusion events associated with fertilization. When the associated double bonds with unsaturated fatty acids deform, the membrane fluidity decreases and it leads to a consequent loss of sperm function. Of note, the lowest results for IVF were manifested after 8 months.

The ability of the embryo to survive appears to be negatively correlated with the level of DNA fragmentation in the germ line (56). Previous studies have shown that DNA-damaged sperm can not fertilize an oocyte (54, 57). Our results in this study have shown that by using sperm from varicocelized rats some of the fertilized eggs stopped at the 2-cell embryo phase. The ultimate outcome of IVF in comparison to the control group was found remarkably low.

## Conclusion

Our data suggest that following varicocele induction severe damage occurs in the process of spermatogenesis which influence produced spermatozoa quality, maturation (testicular and epididimal) and DNA integrity. On the other hand, increasing impaired and abnormal sperm leads to remarkable ROS stress that can result in sperm plasma membrane disintegrity. Ultimately these impairments affect sperm capacitation. It may be concluded that for IVF purposes either sperm samples from varicocele patients should not be used or sperm samples that are collected early could be used after testing for DNA integrity.
